# Consensus on recommended evaluation tools in multiple sclerosis (CORE-MS): A Delphi study protocol on balance and gait assessment

**DOI:** 10.1371/journal.pone.0337440

**Published:** 2026-01-02

**Authors:** Rebecca Cardini, Elisa Gervasoni, Marco Germanotta, Irene Giovanna Aprile, Francesca Cecchi, Chiara Pedrini, Massimiliano Gobbo, Joel Pollet, Rosa Pullara, Maria Pia Amato, Guido Pasquini, Filippo Gerli, Davide Cattaneo

**Affiliations:** 1 IRCCS Fondazione Don Carlo Gnocchi, Milan, Italy; 2 IRCCS Fondazione Don Carlo Gnocchi, Florence, Italy; 3 Department of Experimental and Clinical Medicine, University of Florence, Florence, Italy; 4 Department of Clinical and Experimental Sciences, University of Brescia, Brescia, Italy; 5 Department of NEUROFARBA, University of Florence, Florence, Italy; 6 Department of Pathophysiology and Transplantation, University of Milan, Milan, Italy; Universita degli Studi di Napoli Federico II, ITALY

## Abstract

**Background and objectives:**

Gait and balance impairments are common and disabling in People with Multiple Sclerosis (PwMS), significantly affecting mobility and quality of life. Although several clinical and instrumental tools exist to assess these functions, there is no consensus on the most appropriate measures for detecting changes at the clinical, movement quality, and neural levels. This study aims to establish expert consensus on the most appropriate tools for assessing gait and balance in PwMS to support an individualized approach to rehabilitation.

**Methods:**

The process will begin with a focus group to gather initial feedback from a few experts, followed by a Delphi study consisting of iterative rounds of anonymous surveys with international experts to reach consensus on the most appropriate tools. The Delphi process will be conducted using an electronic platform to ensure anonymity and international participation. Experts will evaluate the proposed tools over several rounds until consensus is reached. The consensus threshold will be predefined, and statistical measures of agreement will guide the analysis.

**Discussion and conclusion:**

By identifying a core set of outcome measures covering clinical, movement quality, and neural aspects, this study aims to address the current fragmentation in clinical practice and research in PwMS. This comprehensive approach will improve the assessment of gait and balance and facilitate the design of tailored rehabilitation interventions that meet the specific needs and recovery potential of each patient. In addition, the study will establish a consensus-based framework for gait and balance assessment in MS rehabilitation, promoting consistency across clinical and research settings. The results are expected to inform future studies on patient stratification, treatment effectiveness, and precision rehabilitation strategies, ultimately leading to improved functional outcomes and quality of care.

## Introduction

Gait and balance impairments are highly prevalent in People with Multiple Sclerosis (PwMS), affecting up to 80% over the course of the disease [1]. These impairments are associated with increased risk of falls, reduced mobility, and decreased quality of life, highlighting the critical need for effective rehabilitation strategies [2,3].

To optimize rehabilitation interventions, according to the patients’ specific needs, a precise and comprehensive assessment is crucial. However, the selection of appropriate assessment tools remains challenging due to the wide variety of measures available [3]. The heterogeneity of assessment tools not only hinders the comparability of findings across different studies, but also prevents the integration of data from multiple studies [4]. To address this gap, appropriate outcome measures at different stages of rehabilitation need to be carefully selected: before and during the intervention (to guide its implementation) and at the conclusion (to assess its efficacy or effectiveness).

In this perspective, a theoretical framework was proposed by Levin et al., which could help in the selection of the most suitable tools [5]. The authors suggested a classification of recovery and compensation which takes into account the Health condition and the first two domains of functioning identified by the International Classification of Functioning (ICF) [6], e.g. Body Functions and Structure and Activity, by defining true recovery as the reappearance of basic motor patterns that were present before the central nervous system injury (the so-called “relearning”), whereas motor compensation is defined as the emergence of new motor patterns resulting from adaptation to the new condition, that is, following the neurological damage (in other words, the learning of new motor strategies).

The authors emphasize the necessity of distinguishing between recovery and compensation following rehabilitation across three distinct levels: the health condition level (i.e., the neural level), the body functions/structure level (i.e., the movement quality level), and the activity level (i.e., the functional level) [5]. Specifically, they advocate for the integration of functional measures alongside impairment-level assessment tools (such as electromyography and kinematic analyses). This approach would facilitate a clearer distinction between true recovery and compensatory mechanisms at the level of basic motor patterns employed.

Indeed, by distinguishing between functional capacity (the ability to successfully complete a task), movement quality (how the task is carried out), and the neural mechanisms (activation of brain areas), clinicians would be better equipped to design personalized treatment plans that focus on either maximizing recovery or promoting compensation, depending on the subject’s specific needs [5]. Although originally developed for post-stroke subjects, this framework can also be applied to other neurological conditions such as multiple sclerosis. A recent study reported that following gait rehabilitation, PwMS showed a more physiological activation impulse during the clearance phase, and exhibited activation profiles more consistent with those observed in healthy subjects, supporting the distinction between true recovery and compensatory strategies [7].

A standardized, evidence-based approach to gait and balance assessment would provide a solid foundation to promote targeted and adaptive rehabilitation, ensuring that rehabilitation strategies are tailored to each patient’s specific needs and recovery potential [8–11].

Expert consensus is crucial for selecting the most informative evaluation tools to advance a precision rehabilitation model that integrates individualized assessments for developing tailored interventions and therefore maximizing functional recovery [12]. In this scenario, the Delphi method is a well-established and appropriate approach to address complex and poorly defined clinical questions [12]. Originally developed by the RAND Corporation in the 1950s [13], the Delphi method is designed to facilitate expert consensus through structured, iterative surveys. It is particularly well suited to synthesizing diverse expert opinions when empirical evidence alone is insufficient to establish best practices [14]. The strengths of the method are the anonymity of panelists (reducing bias), controlled feedback (ensuring informed decision-making), and iterative rounds (refining expert opinion), making it an ideal choice to identify the most appropriate rating scales and tests for the assessment of gait and balance in PwMS [15]. In addition, the adaptability of the Delphi process to electronic formats (E-Delphi) allows for the inclusion of geographically diverse expert panels and accelerates the consensus-building process [14].

On this basis, the present study will use a Delphi panel to identify the best evidence-based tools for assessing gait and balance in PwMS at the clinical, movement quality, and neural levels. By establishing expert consensus, we aim to provide clinicians and researchers with a robust foundation for improving the assessment and management, ultimately contributing to enhanced, individualized rehabilitation strategies for PwMS.

## Materials and methods

### Study design

We plan to conduct a focus group as a pilot test, followed by a Delphi panel. The focus group will involve a small group of panelists, similar to those invited to the Delphi study, to ensure clarity in the data collection process, instructions, and questions [16]. This preliminary step will also help to identify national and international panelists. Additionally, the team will ask prospective panelists to share their thoughts on the study topic in an open-ended format prior to the first round of structured questions.

The Delphi study consists of iterative rounds of standardized surveys with a selected group of anonymized panelists, aimed at reaching expert consensus on a given topic [12]. The Delphi method is widely used to develop standards, measurement tools, guidelines, and expert recommendations. It has also been successfully used to develop consensus definitions for terms that lack clear or universally accepted interpretations, making it an invaluable tool for advancing clinical practice in areas that require further clarity and agreement [14,15,17–21].

Thus, the focus group and Delphi panel methodologies are well suited for generating information and building consensus on clinical tools used to assess balance and gait in PwMS across all clinical forms of the disease—an area that requires further refinement and standardisation.

### Expert focus group methodology

We will begin by conducting a focus group comprised of highly experienced clinicians, researchers, and engineers. We plan to recruit between 4 and 12 participants, in line with existing qualitative research guidelines [22,23], to explore the best assessment tools for evaluating gait and balance in MS based on the theoretical framework by Levin et al. [5]. Results will be anonymized to encourage honest responses, and the findings will be used to set up the Delphi study [16].

**Fig 1:**
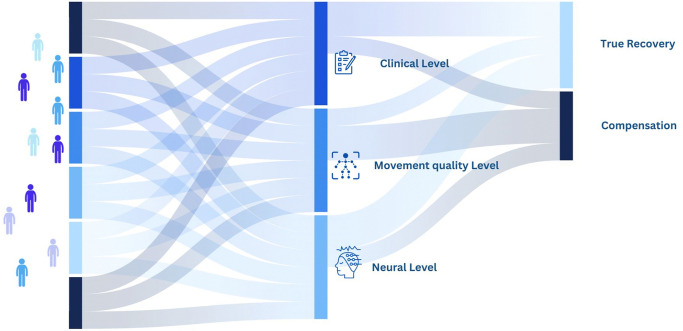
Conceptual framework of the expert focus group methodology.

According to the ICF framework (activity, body functions, and body structures), the focus group will be asked to answer questions such as: “What is the core set of scales and tests to assess true recovery and compensation at a clinical level, i.e., movement quality of the task/activity, using the same end effectors and joints used by non-disabled individuals?”; “What is the core set of instruments to evaluate true recovery and compensation at the movement quality level, i.e., movement pattern?”; “What is the core set of tools to assess true recovery and compensation at the neural level, i.e., neural activation?” (Fig 1**).**

Once all responses are collected, the results will be presented as a starting point for further discussion, allowing participants to elaborate on their views. Finally, based on our literature search, we will provide a list of existing assessment tools and ask the focus group to anonymously select the most relevant ones. Once all responses are recorded, the results will be presented and discussed.

### Delphi methodology

Using the data from the focus group and the existing literature on this topic, we intend to formulate relevant questions about the assessment tools for evaluating gait and balance in PwMS at the three levels. To ensure methodological rigor and neutrality throughout the Delphi process, a facilitator will be appointed. The facilitator’s role is purely procedural and includes designing and distributing questionnaires, summarizing responses, and providing anonymized feedback between rounds, without influencing expert judgments. Neutrality is maintained through participant anonymity, structured and aggregated feedback, and the transparent application of predefined procedures. We will use an electronic version of the Delphi study (E-Delphi) [13], which allows panelists to engage in discussions while maintaining anonymity. This platform automates data analysis and the preparation of individualised reports for each panelist, helping to expedite data collection and reduce the costs associated with convening large-scale national and international panels [24]. An easy-to-use E-Delphi platform will be employed for data collection. These platforms enable efficient survey distribution, real-time data analysis, and improved participant engagement. Their use also minimizes logistical challenges and accelerates survey rounds [14].

### Delphi experts’ recruitment

According to the guidelines, we will involve between 30 and 50 participants [14]. We will recruit more panelists than the minimum required to allow for potential attrition. The expert panel will consist of clinicians (medical doctors and occupational and physical therapists), researchers, engineers, and professionals with expertise in gait, balance, and MS rehabilitation. Recruitment will prioritize heterogeneity in both professional background and geographical representation to ensure a comprehensive range of perspectives. Panelists will be purposively selected to ensure they have the necessary expertise and experience, with criteria including proven or self-identified expertise in the topic, institutional affiliation, peer nominations, and previous publications in the field [25]. We will use a multi-pronged approach, including direct outreach to subject matter experts identified by the team. To facilitate engagement and promote transparency, individual feedback reports will be provided to panelists, highlighting how their responses compare to those of other participants in the previous round. To minimize attrition, we intend to acknowledge panelists and offer co-authorship on manuscripts derived from the study findings. We will also clearly communicate the aims and methods of the study and keep panelists informed of the research process and any changes to the timeline.

### Delphi administration

**Fig 2:**
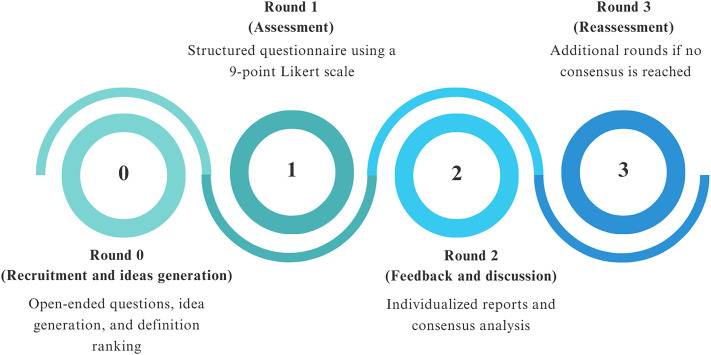
E-Delphi rounds.

The Delphi study will consist of four rounds, conducted via an e-Delphi platform to enable global participation and streamline data collection. Panelists will have between one and four weeks to complete each round. Each round will be structured as follows (Fig 2):

- *Round 0 (Recruitment and ideas generation)* – We will collect data on panelists’ expertise and years of experience. Open-ended questions will be used to gather initial insights into appropriate assessment tools, and to allow them to suggest changes to the definitions presented. Panelists should also provide comments and explanations for their answers.
- *Round 1 (Assessment)* – We intend to synthesize the panelists’ input from Round 0 and create a close-ended structured questionnaire for Round 1, in which we will ask participants to indicate which tools they consider most appropriate for assessing balance and gait in PwMS [13].
- *Round 2 (Feedback and discussion)* – Panelists will receive individualized reports showing how their responses to Round 1 questions compare with those of other panelists, and whether the panel reached a consensus. They will be asked to rate their level of agreement using a 9-point Likert scale (1 = strongly disagree; 9 = extremely agree). An option for missing responses (“unable to rate”) will also be provided.
- *Round 3 (Reassessment)* – If a consensus is not achieved after the second round, up to three rounds will be conducted. The suggested changes from the open-text field may be used to make changes.

### Consensus definition

The primary outcome of interest will be the consensus on the definition of a core-set assessment for balance and gait at the clinical, movement quality, and neural levels. Consensus will be defined as agreement among ≥ 75% of panelists, a threshold commonly reported in Delphi studies (range 50–97%) [15,26], and this will serve as the a priori criterion for this study. Measures of central tendency and dispersion, such as medians and interquartile ranges, will be used to quantify agreement. If consensus on a definition is not reached, we will report the results of each round, explore reasons for the lack of consensus, and narratively discuss the implications of the findings. Anonymity will be guaranteed for all panelists, but not for the research team.

### Delphi response analysis

We will analyze the results of each Delphi round separately, using descriptive analysis to report frequencies and percentages. This will help to assess the responses from each round and determine the level of consensus for each item.

### Study schedule

**Fig 3:**
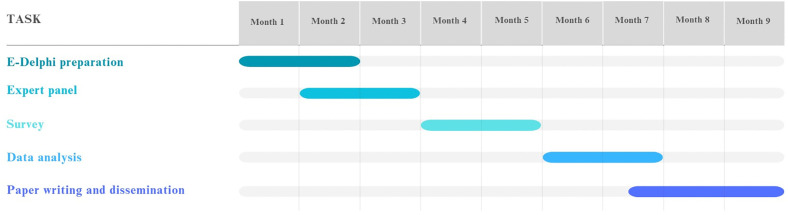
E-Delphi project timeline for 2025-2026.

Preparation for the e-Delphi process will begin in June 2025 and continue through the end of July. Expert recruitment will start in early July 2025 and run until the end of August. The online survey will take place from September to the end of October 2025. We anticipate completing data analysis in December 2025 and manuscript preparation by the end of February 2026 (Fig 3).

### Ethical considerations

Ethical review and approval were not required for the study on human participants in accordance with the local legislation and institutional requirements. Written informed consent from the participants was not required to participate in this study in accordance with the national legislation and the institutional requirements.

### Knowledge translation

The research team has worked with clinicians, researchers, engineers, and professionals with expertise in gait, balance, and rehabilitation of PwMS to discuss the rationale and methodology of the study and to identify the key theoretical and methodological challenges that we intend to address. We will disseminate the results of the study through publication in a journal focused on rehabilitation research to ensure that the results reach the appropriate stakeholders. We also plan to submit the results for presentation at national and international conferences. The team will receive feedback on the definitions developed from theoretical, methodological, and clinical perspectives, which will guide future research efforts.

## Discussion

The assessment of gait and balance in PwMS requires an evidence-based approach to ensure accurate evaluation and effective rehabilitation. Due to the complexity and multidimensional nature of these assessments, expert consensus is crucial in selecting the most appropriate tools. However, despite the availability of various assessment scales and tests, there is no established consensus on which are best suited to evaluate gait and balance across clinical, movement quality, and neural levels. The lack of common outcome measures, according to a specific framework, makes it difficult to compare studies, monitor disease progression, and design targeted rehabilitation programs. A shared assessment framework is essential not only for improving clinical decision-making but also for better and more comprehensive understanding of study results in terms of recovery versus compensation and to guide rehabilitation strategies.

Rehabilitation research increasingly recognizes the importance of structured models to characterize recovery and compensation mechanisms. Hart et al. [27] proposed the Rehabilitation Treatment Specification System, which provides a structured approach to defining rehabilitation interventions based on their goals, ingredients, and mechanisms of action leading to true recovery or compensatory strategies. This framework ensures that rehabilitation strategies are based on clearly identified treatment targets, helping clinicians move beyond symptom-based approaches and toward interventions grounded in an understanding of motor control mechanisms typically assessed not only with tools measuring function but by integrating results from these tools with data exploring movement quality (movement quality level) and neural changes (neural level). Standardised assessment tools aligned with such frameworks would facilitate the stratification of patients based on their functional characteristics, supporting precision rehabilitation approaches that optimize recovery or compensation strategies when needed. Beyond its clinical implications, this study also contributes methodologically to rehabilitation research. While the Delphi method has been widely used in healthcare for developing guidelines and reaching expert consensus, its application in rehabilitation remains limited, with only a small number of studies using this approach in neurological disorders [18,17].

Establishing a structured study protocol allows us to provide a replicable methodology that can be used in other areas of rehabilitation where a lack of consensus hinders progress. To our knowledge, this is one of the first Delphi studies focused on standardizing gait and balance assessment in PwMS rehabilitation, and we hope it will pave the way for broader applications of consensus-driven approaches in the field.

## Conclusion

This study aims to establish expert consensus on the best assessment tools for gait and balance in PwMS, ensuring a standardised approach according to a specific framework that include the evaluation of recovery and compensation at the clinical, movement quality, and neural levels. This approach would enhance the use of identified outcome measures in both clinical practice and research. Our Delphi study will help define future research, including a cross-sectional study to assess the prevalence of gait and balance impairments and to classify PwMS into clinically meaningful subgroups, helping to refine precision rehabilitation approaches. Results from this study will also be useful in longitudinal studies examining the evolution of gait and balance rehabilitation, linking changes in clinical, movement quality, and neural outcomes to specific therapeutic interventions.

In conclusion, the consensus developed through this Delphi process will provide a solid basis for precision rehabilitation in PwMS, ultimately improving rehabilitation strategies to maximize functional recovery.
